# Heightened eating drive and visual food stimuli attenuate central nociceptive processing

**DOI:** 10.1152/jn.00504.2014

**Published:** 2014-12-04

**Authors:** Hazel Wright, Xiaoyun Li, Nicholas B. Fallon, Timo Giesbrecht, Anna Thomas, Joanne A. Harrold, Jason C. G. Halford, Andrej Stancak

**Affiliations:** ^1^Department of Psychological Sciences, University of Liverpool, Liverpool, United Kingdom; and; ^2^Unilever R&D, Vlaardingen, The Netherlands

**Keywords:** EEG, source analysis, pain, hunger, appetite

## Abstract

Hunger and pain are basic drives that compete for a behavioral response when experienced together. To investigate the cortical processes underlying hunger-pain interactions, we manipulated participants' hunger and presented photographs of appetizing food or inedible objects in combination with painful laser stimuli. Fourteen healthy participants completed two EEG sessions: one after an overnight fast, the other following a large breakfast. Spatio-temporal patterns of cortical activation underlying the hunger-pain competition were explored with 128-channel EEG recordings and source dipole analysis of laser-evoked potentials (LEPs). We found that initial pain ratings were temporarily reduced when participants were hungry compared with fed. Source activity in parahippocampal gyrus was weaker when participants were hungry, and activations of operculo-insular cortex, anterior cingulate cortex, parahippocampal gyrus, and cerebellum were smaller in the context of appetitive food photographs than in that of inedible object photographs. Cortical processing of noxious stimuli in pain-related brain structures is reduced and pain temporarily attenuated when people are hungry or passively viewing food photographs, suggesting a possible interaction between the opposing motivational forces of the eating drive and pain.

hunger and pain have basic homeostatic components, and both necessitate alleviative actions. When experienced singularly the course of action is obvious, but when experienced together the appropriate response may be less apparent. If the hunger drive is present but weak, it should be possible to ignore it and escape from the pain. If mild pain is present but the hunger drive is stronger, it should be possible to eat while ignoring pain. If either drive is sufficiently pressing, the other may not even receive conscious consideration.

Abundant evidence shows that eating (Aloisi and Carli 1996; Casey and Morrow 1983; Foo and Mason 2005; Kakeda et al. 2010; LaGraize et al. 2004; Schobel et al. 2012; Segato et al. 1997; Zmarzty et al. 1997), or expecting to eat (Dum and Herz 1984), can reduce pain. This effect is present in even very young infants, both animal (Blass et al. 1987, 1991; Blass and Fitzgerald 1988; Blass and Shide 1994; Ren et al. 1997; Shide and Blass 1989) and human (Blass and Hoffmeyer 1991; Blass and Shah 1995; Bucher et al. 1995; Lehr et al. 2014; Shah et al. 2012; Yilmaz et al. 2014). The antinociceptive effect of feeding may be at least partly attributable to activation of vagal nerve afferents as the stomach stretches; this inhibits perception of several types of pain (Faris et al. 2006; Sedan et al. 2005). Conversely, a smaller body of evidence shows that fasting can produce analgesia (Davidson et al. 1992; de los Santos-Arteaga et al. 2003; McGivern et al. 1979; McGivern and Berntson 1980). Interestingly, fasting is employed as a treatment for chronic pain in some settings (Michalsen et al. 2002, 2005; Michalsen and Li 2013; Wilhelmi de Toledo et al. 2013).

One factor playing a role in fasting- or feeding-related analgesia might be the endogenous opioid system, as studies that administered naltrexone or naloxone found that the analgesia effect was reversed (Blass et al. 1987, 1991; Blass and Fitzgerald 1988; Davidson et al. 1992; de los Santos-Arteaga et al. 2003; Dum and Herz 1984; McGivern et al. 1979; McGivern and Berntson 1980; Segato et al. 1997; Shide and Blass 1989). Such compounds also reduce the pleasure normally derived from ingesting palatable foods (Drewnowski et al. 1995; Fantino et al. 1986; Halford et al. 2010; Schneider et al. 2010). Endogenous opioids are intricately involved in feeding regulation (see Bodnar 2004 for a comprehensive review), and levels of β-endorphins rise when eating highly palatable food (Dum et al. 1983; Mercer and Holder 1997). Fasting induces a decrease in endogenous opioid levels but an increase in β-hydroxybutyrate (Pan et al. 2000), an isomer of the illicit drug γ-hydroxybutyrate (Brown 2007). γ-Hydroxybutyrate produces a considerable rise in dopamine release (Gessa et al. 1966; Itzhak and Ali 2002). Dopamine appears to play a significant role in analgesia (Jarcho et al. 2012; Wood 2006) and reductions in affective responses to pain (Jarcho et al. 2012; Tiemann et al. 2014), and enhanced dopamine levels are diminished by naloxone (Klitenick et al. 1992; Seilicovich et al. 1985; Snead and Bearden 1980). The evidence reviewed above suggests that both physiological and hormonal factors play a part in food-related analgesia.

If hunger can override pain and vice versa, a mechanism can be postulated that allows attending to one of these drives at the expense of the other. Pain, feeding, and taste pathways are represented in the brain stem (Craig 2003; Grill and Norgren 1978; Small et al. 2003), hypothalamus (Burton et al. 1976; Dafny et al. 1996; Guyton and Hall 2004), and amygdala (Bernard et al. 1992; Bernard and Besson 1990; Neugebauer and Li 2002; Sanghera et al. 1979). Additionally, the pain and taste pathways are represented in the primary somatosensory cortex (Gingold et al. 1991; Kenshalo et al. 1980; Norgren 1990), anterior insula (Dostrovsky and Craig 1996; Pritchard et al. 1986; Scott et al. 1986; Sudakov et al. 1971), and anterior cingulate cortex (ACC) (Bushnell and Duncan 1989; Hutchison et al. 1999; Sikes and Vogt 1992). Any of these areas could therefore facilitate a competitive interaction between hunger and pain.

Rewarding stimuli other than food, such as monetary gain (Becker et al. 2013) and the expectation of analgesia (Elsenbruch et al. 2012), have also been shown to inhibit the perception of pain. According to the motivation-action theory of emotion (Bradley et al. 2001; Lang et al. 1992, 1997), two motivational systems are posited to exist, one appetitive, the other defensive. The appetitive system is engaged by approach-related reinforcers such as ingestion; the defensive system is triggered by threat.

The aim of this study was to explore competitive interactions between the appetitive and defensive motivational systems, using fasted vs. fed states and presenting food photographs in combination with painful laser stimuli. We hypothesize that viewing food cues should activate the appetitive system, inhibiting the pain-activated defensive system and resulting in a suppression of subjective pain perception and pain-related brain activity. Provided that the visual food cues are sufficiently motivationally salient, such a pain decrease is also predicted by the motivation-decision model of pain modulation (Fields 2007), whereby endogenous opioids suppress responses to noxious stimuli to allow response to more important motivational stimuli. Such a reciprocal relationship between pain and other motivational stimuli, such as fear and social cues, has already been described (Wiech and Tracey 2013), but this is the first study to investigate brain areas involved in facilitating competition between the motivational drives of appetite and pain with source reconstruction of laser-evoked potentials (LEPs).

## METHODS

### 

#### Participants.

Fourteen healthy volunteers (7 men, 7 women) with a normal body mass index (BMI; World Health Organisation 2006) from the undergraduate and postgraduate student population of the University of Liverpool took part in this study. The average age of the participants was 24.6 ± 4.1 yr (mean ± SD). Participants gave their written informed consent, and the study was conducted in accordance with the Declaration of Helsinki. Ethical approval was obtained from the University of Liverpool Research Ethics Committee.

#### Procedure.

Participants were asked on the day before both sessions not to exercise more than they would normally and not to eat or drink anything other than water from midnight. Compliance was assessed with diary entries; no participants reported taking part in any strenuous exercise or excessive eating. Participants completed two sessions, which were separated by an average of 7.5 days (±3.6), and attended the lab at 8:45 AM after a 9-h overnight fast. Participants either remained fasted for the remainder of the session (fasted condition) or were provided with a standardized breakfast (fed condition). Session order was counterbalanced across participants. The breakfast consisted of cornflakes, semiskimmed milk, toast, margarine, jam, orange juice, and tea/coffee. The total energy content was 531 kcal (2,223 kJ) for women and 680 kcal (2,847 kJ) for men. Measures of hunger, desire to eat, and prospective consumption (how much food could potentially be eaten) were taken with 100-mm visual analog scales (VAS) immediately prior to the EEG recording in both the fasted and fed sessions.

The experiment took place in a sound-attenuated room. Painful stimuli were produced with an Nd-YAP laser stimulator (Stim1340, El.En.); the spot size was 5 mm, and the pulse duration was 3 ms. Before the experiment began a 5-cm circle was drawn on the dorsal surface of the participants' right hand, and the laser stimuli were applied pseudorandomly within. The laser intensity to be used was selected by applying a succession of painful stimuli ranging from 1.0 J up to 2.25 J. Intensities started off low, and after each stimulus the participant was asked to rate, on a scale of 1 to 7, how much pain he/she had felt. The scale was anchored as “1: no pain” and “7: worst imaginable pain.” The laser intensity was gradually increased until participants gave a rating of 3–4 (moderate pain). When this rating was achieved, three more stimuli at this intensity were applied to ensure that the rating remained consistent. Participants were given five practice trials and shown how to make their response.

**Fig. 1.**
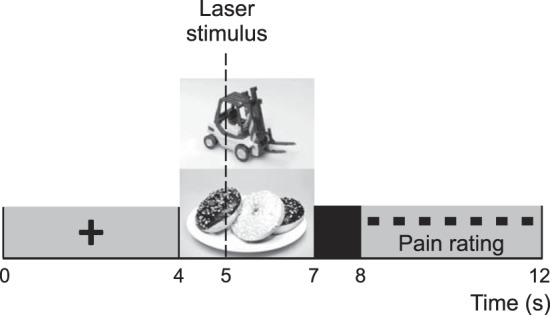
Trial structure. Each trial began with a fixation cross 4,000 ms long, which was replaced by a photograph of either food or an inedible object. The photograph was visible for 3,000 ms, and a laser stimulus was applied exactly 1,000 ms after the onset of the photograph. The screen went blank for 1,000 ms, and then participants rated the pain they had felt on a visual analog scale of 1–7 (anchored as 1 = no pain, 7 = worst imaginable pain). The scale was incremented by repeatedly clicking the mouse. Equal numbers of food and object photographs were presented within each block.

The experiment was comprised of three blocks, each containing 32 trials. Figure 1 shows a visual representation of the trial structure. We divided the experiment into blocks for reasons of safety and data quality. As upwards of 90 laser stimuli were applied to the dorsum of the hand, it was prudent to check for skin burns or abnormal skin reactions between successive blocks of stimuli. The stimulation period took ∼40 min, and therefore, to avoid fatigue and any discomfort due to limited mobility, resting periods also served to allow participants to stretch and relax. Finally, the electrode sponges required regular remoistening in order to keep electrical impedance low. The approximate amount of time between blocks ranged from 4 to 8 min, depending on the time it took to resaturate the electrodes and/or adjust the laser intensity if participants' pain ratings had noticeably decreased.

The food photographs used in the experiment were selected on the basis of an unpublished pilot study, which had found them to be consistently rated as tasty, enjoyable, and filling. Object photographs (included as the control condition) were a mixture of household and leisure items, unrelated to food or eating. Photographs were 492 × 329 pixels, with a resolution of 72 dpi. Participants were seated 60 cm away from the 19-in. LCD monitor. Delphi 7 (Borland Software, Austin, TX) was utilized as the stimulus presentation program.

At the end of each experimental session, participants rated each food in the photographs for how tasty, filling, and enjoyable it looked, along with how much they would eat of it if they were hungry. Ratings were made on numeric rating scales from 0 to 10.

#### Recordings and data analysis.

EEG was recorded continuously with the 128-channel Geodesics EGI System (Electrical Geodesics, Eugene, OR). The anatomical landmarks of the nasion, inion, and left and right preauricular points were used to position the sensor net. Electrode-to skin-impedances were kept below or equal to 50 kΩ and checked between each block. If necessary, the electrode sponges were remoistened. The band-pass filter was 0.1–200 Hz, and the sampling rate was 1,000 Hz. Electrode Cz was used as the reference. BESA 6.0 (MEGIS) was used for data processing. Raw data were transformed into reference-free data by common average reference (Lehmann 1987). The data were filtered from 0.5 to 70 Hz, with an additional notch filter applied at 50 Hz. Oculographic and electrocardiographic artifacts were removed by principal components analysis (Berg and Scherg 1994), and the data were visually inspected to check for other artifacts. Trials contaminated by additional artifacts were excluded from the analysis.

To analyze the typical period of nociceptive processing the data were divided into epochs from −100 to +600 ms relative to the onset of the laser stimulus, with −100 to 0 ms used as the baseline. LEPs were computed separately for food and object photographs by averaging relevant trials first across single blocks and then averaging the block averages across single sessions. This yielded, for each participant, four overall LEPs: one when hungry and viewing food photographs, one when hungry and viewing object photographs, one when fed and viewing food photographs, and one when fed and viewing object photographs. The average numbers of trials accepted per participant for these four conditions were (of a possible 48) 38.3 ± 8.1, 37.4 ± 8.8, 38.5 ± 6.1, and 37.3 ± 6, respectively. Finally, the individual participant averages were combined to produce group-level condition averages. Additionally, to explore the group-level effects of the appetite manipulation independently of the photograph type, all subjects' hungry condition trials were collapsed across photograph types to produce averaged LEPs for the hungry condition and all subjects' fed condition trials were collapsed across photograph types to produce averaged LEPs for the fed condition.

#### Source dipole reconstruction.

After calculation of the subject- and group-level averaged potentials, sources were estimated from the group-level averaged waveforms with classical LORETA analysis recursively applied (CLARA; Hoechstetter et al. 2010), which takes as a first step a regularized low-resolution electromagnetic tomography (LORETA; Pascual-Marqui et al. 1994) image and then, in iterative steps, smooths the previous image and sets to zero all voxels with amplitudes of <10% of the maximum activation, effectively eliminating them from the analysis. This produces an image containing an amplitude of activation defined for each voxel. A LORETA image taking this voxel-specific information into account is then computed.

Parameters specified in the present CLARA analysis were singular value decomposition (SVD) regularization with a cutoff of 0.01%, two iterations, and a voxel size of 7 × 7 × 7 mm^3^. A significant advantage of using CLARA is that the elimination of voxels with small amplitudes gives rise to a far clearer and more circumscribed image than some other source analysis techniques, making it less problematic to visualize sources that are spatially close. The spatial maxima of activation clusters produced by CLARA were used as seeds for fitting a set of equivalent current dipoles. The orientations of equivalent current dipoles were fitted separately in both grand average and individual LEPs.

The four-shell ellipsoid head model was employed, using the following conductivities (S/m = Siemens per meter): brain = 0.33 S/m; scalp = 0.33 S/m; bone = 0.0042 S/m; cerebrospinal fluid = 1.0 S/m.

#### Statistical analysis.

Unless otherwise stated, SPSS v. 20 (IBM) was utilized to perform statistical analyses. Repeated-measures ANOVAs were used to test for the effects of sessions, photographs, and blocks on the source dipole waveforms and pain ratings. Paired Student's *t*-tests were used to compare sessions and photographs. All *P* values were Bonferroni corrected in order to account for multiple comparisons, and a 95% confidence level was employed throughout. Pearson's correlation coefficients were computed to test for correlations between source dipole moments and various self-report measures. Differences between correlation coefficients were evaluated with Statistica 7.0 (StatSoft, Tulsa, OK).

Because of the absence of previous studies addressing the effects of appetite on LEPs, we did not identify any specific LEP component or latency period of interest a priori. Instead we employed a data-driven analysis, which allowed the detection of clusters of signals and intervals of interest manifesting the effects of one or more independent variables and their interaction. To this end the source dipole waveforms were analyzed by repeated-measures ANOVA and a random permutation test (Maris and Oostenveld 2007) in MATLAB v. 7.8 (MathWorks, Natick, MA), using 1,000 permutations and a 99% confidence level. The permutation method was necessary to control for the inflated risk of type I error, brought about by the large number of ANOVA tests required.

## RESULTS

### 

#### Behavioral data.

The goal of giving participants a large breakfast was to ensure that they felt full and not hungry, leading to clearly delineated hungry and fed conditions. Paired *t*-tests were employed to compare the level of eating drives between sessions. When participants were fed compared with fasting, VAS ratings were significantly lower for hunger [mean ± SD for hungry session: 62.79 ± 15.5, for fed session: 9.43 ± 15.8; *t*(13) = 11.1, *P* < 0.001], desire to eat [mean ± SD for hungry session: 64.93 ± 19.6, for fed session: 8.79 ± 11.8; *t*(13) = 9.9, *P* < 0.001], and prospective consumption [mean ± SD for hungry session: 57.79 ± 18.3, for fed session: 15.86 ± 16.3; *t*(13) = 8.0, *P* < 0.001].

Laser intensities were necessarily adjusted between blocks for most of the participants, because of them becoming habituated to the laser and reducing their subjective responses to the noxious stimuli. Collapsing the laser intensities across blocks revealed that the laser intensity was almost identical in both conditions; the mean intensity used was 2.45 ± 0.35 J in the hungry condition and 2.47 ± 0.31 J in the fed condition. This produced a mean intensity difference of 0.02 J between conditions, which was statistically not significant [*t*(13) = −1.1, *P* = 0.298].

**Fig. 2.**
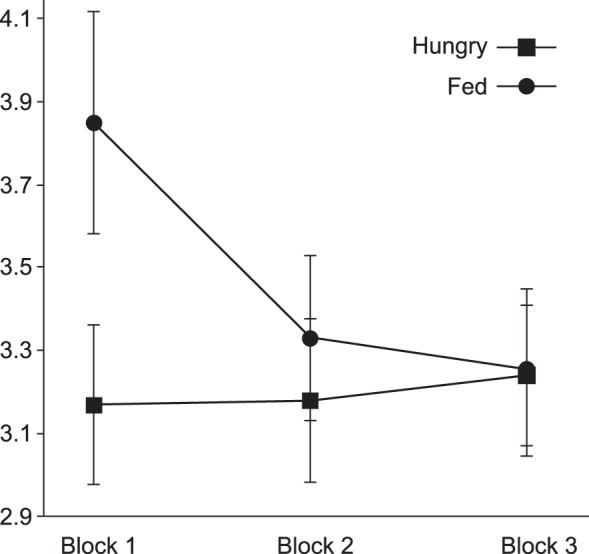
Mean ± SD pain intensity ratings during the hungry and fed sessions, averaged by block. There is a significant difference in *block 1* between the mean pain ratings in the hungry session (3.17 ± 0.74) compared with the fed session (3.85 ± 1.0).

Pain ratings were analyzed with a 2 (sessions) × 2 (photographs) × 3 (blocks) ANOVA for repeated measures. Mean pain ratings did not significantly differ between the sessions (hungry and fed, collapsed across all photographs and all blocks), nor were they statistically different across the two photograph types (collapsed across all blocks). There was, however, a statistically significant effect of blocks on mean pain ratings [*F*(2,26) = 4.49, *P* < 0.05] and a statistically significant interaction between session and blocks [*F*(2,26) = 3.72, *P* < 0.05]. As shown in Fig. 2, an analysis of simple effects revealed that pain ratings in the fed session in *block 1* were significantly larger than those in *blocks 2* [*t*(13) = 3.4, *P* < 0.05] and *3* [*t*(13) = 3.1, *P* < 0.05]. *Blocks 2* and *3* were not significantly different from each other. There was a statistically significant difference between the pain ratings during *block 1* across sessions [*t*(13) = 2.16, *P* < 0.05].

Student's paired *t*-tests were used to compare the food photograph ratings obtained after both EEG recordings. There were no statistically significant differences between the sessions (*P* > 0.05).

#### Source dipole model of LEPs.

**Fig. 3.**
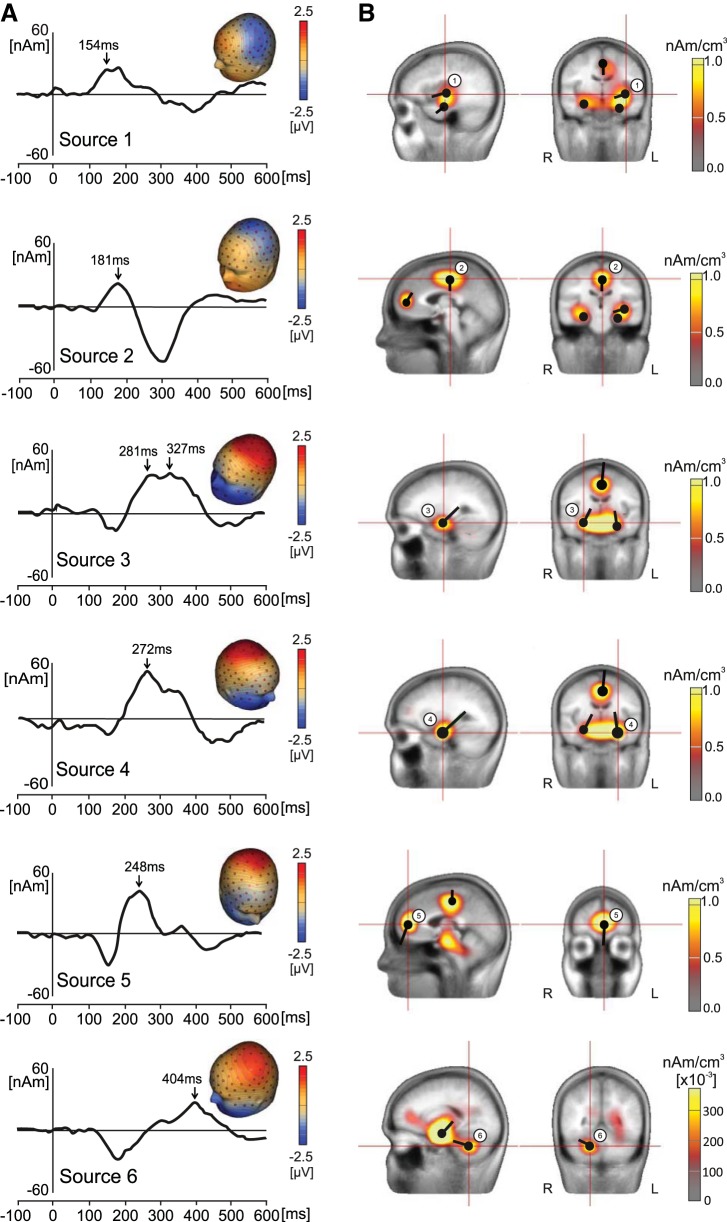
*A*: waveforms of *sources 1–6*, collapsed across both sessions (hungry and fed) and both photograph types. Peak latencies are denoted by an arrow. *B*: *sources 1–6* superimposed over a standard structural MRI scan. In each image, the source corresponding to the waveform in *A* is indicated by its number and by the red cross hairs.

Figure 3*A* shows the grand averaged waveforms and topographic maps of LEPs at different equivalent current dipoles localized with CLARA, on data combined from all sessions, photographs, and blocks. Figure 3*B* illustrates the spatial clusters obtained in CLARA analysis and locations of equivalent current dipoles that were fitted based on CLARA. The source model of the LEPs accounted for 94.34% of the variance. It was best constructed by six source dipoles; adding another source did not explain significantly more of the variance. Tables 1–3 show anatomical labels and approximate Talairach coordinates of each of the six source dipoles.

**Table 1.** Source significantly modulated by session

Anatomical Label	Talairach, mm	Epoch, ms	Hungry Mean ± SD	Fed Mean ± SD	*F*	*P*
Right PHG (*3*)	27, −9, −16	248–257	16.32 ± 23.0	27.51 ± 21.9	5.10	0.042

The anatomical label of the source (source number shown in parentheses) with corresponding approximate Talairach coordinates (*x*, *y*, *z*) and time epoch of significant modulations are shown. Mean values refer to the mean amplitude of the source waveform during the time interval when it was significantly modulated by a session. PHG, parahippocampal gyrus.

**Table 2.** Sources significantly modulated by photograph type

Anatomical Label	Talairach, mm	Epoch, ms	Food Mean ± SD	Object Mean ± SD	*F*	*P*
Left OIC (*1*)	−37, −12, −2	150–160	19.30 ± 19.3	21.92 ± 18.2	7.18	0.019
ACC (*2*)	−3, −21, 45	167–177	9.79 ± 13.0	13.00 ± 13.2	7.06	0.020
Right PHG (*3*)	27, −9, −16	300–330	27.75 ± 13.9	35.00 ± 14.5	16.08	0.001
Cerebellum (*6*)	18, −50, −35	395–405	14.00 ± 10.8	19.61 ± 13.3	5.18	0.040

The anatomical labels of the sources (source numbers shown in parentheses) with corresponding approximate Talairach coordinates (*x*, *y*, *z*) and time epochs of significant modulations are shown. Mean values refer to the mean amplitude of the source waveform during the time interval when it was significantly modulated by a session. “Food” and “Object” refer to the food and object photograph types. OIC, operculo-insular cortex; ACC, anterior cingulate cortex.

**Table 3.** Sources not significantly modulated by either experimental manipulation

Anatomical Label	Talairach, mm	Epoch	Hungry/Food	Fed/Object	*F*	*P*
Left PHG (*4*)	−27, −9, −21	—	—	—	—	—
MFG (*5*)	−3, 49, 8	—	—	—	—	—

This table shows sources that were not significantly modulated during any time epochs by either experimental manipulation but still contributed significantly to the source dipole model. The anatomical labels of the sources (source numbers shown in parentheses) with corresponding approximate Talairach coordinates (*x*, *y*, *z*) are shown. “Food” and “Object” refer to the food and object photograph types. MFG, middle frontal gyrus.

*Source 1* was tangentially oriented and located in left operculo-insular cortex. Its shortest latency peak occurred at 154 ms, with a negative maximum over the temporal electrodes. Both the topographic pattern and the peak latency suggest that this component is equivalent to the N1 component of LEPs. *Source 1* peaked again at 188 ms (topographic map not shown), during the period in which the N2 component is known to operate. *Source 2* was associated with a strong negative scalp potential field at the vertex peaking at 181 ms, corresponding to the N2 component of LEPs. It was a radially oriented dipole located in the anterior mid-cingulate cortex. *Sources 3* and *4* were located in bilateral parahippocampal gyri (PHG). *Source 3* showed two peaks, one at 281 ms (topographic map not shown) and one at 327 ms; *source 4* peaked once at 272 ms. They were obliquely oriented and pointed posteriorly toward the positivity at posterior parietal electrodes. *Source 5* was fitted in the right middle frontal gyrus (MFG) and accounted for a negativity seen in the right frontal electrodes at 248 ms. *Sources 3*, *4*, and *5* contributed to the large P2 LEP component. Finally, CLARA also identified the presence of a comparatively weak source in the right cerebellum, peaking at 404 ms. *Source 6* had a tangential orientation and explained part of the negative spatial maxima seen in electrodes placed on the lower face. The peak latency of *source 6* would correspond to the P2 or N3 component of LEPs (Stancak et al. 2013). Although one previous paper from our laboratory has indicated the presence of a cerebellar component in LEP data (Stancak and Fallon 2013), this source dipole will need further independent confirmation, and therefore any conclusions based on modulations of *source 6* should be treated with caution.

#### Effects of sessions and photographs.

**Fig. 4.**
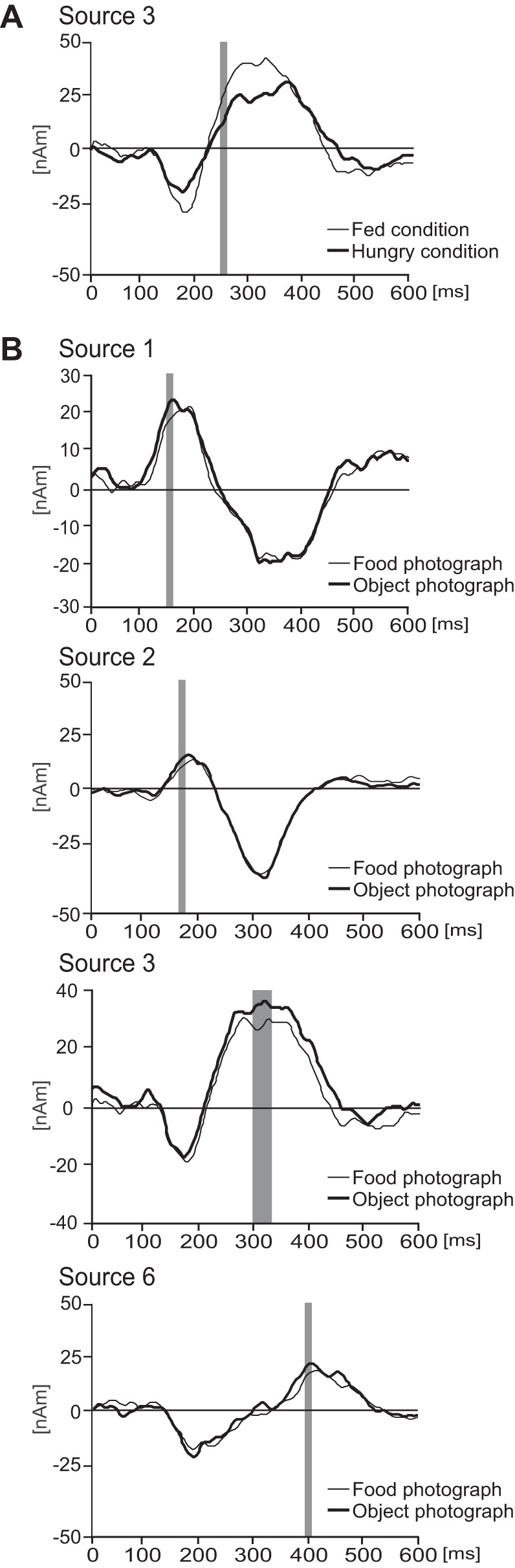
Source waveforms showing modulation by session (*A*) and photograph type (*B*). Time epochs of significant modulations are indicated by gray bars. These epochs were derived from the statistical analysis of the entire laser-evoked potential (LEP) waveform in each condition.

To test the effects of sessions and photographs on LEPs, a two-way ANOVA for repeated measures was carried out for all six waveforms over the entire time interval from −100 to 600 ms. To reduce the chances of a type I error, a permutation analysis (Maris and Oostenveld 2007) was carried out. Figure 4*A* shows the only statistically significant effect of session, which was seen in *source 3*, located in the right PHG. In the time interval of 248–257 ms, the source activity was stronger in the fed session than in the hungry session. Mean values of *source 3* amplitudes in the hungry and fed sessions, as well as *F* and *P* values, are given in Table 1. Figure 4*B* shows the statistically significant effects of photographs on the waveforms of *sources 1*, *2*, *3*, and *6*. Each of these sources showed somewhat smaller activations in the context of food photographs compared with object photographs. Although the statistically significant effects of photograph type were evident from 150 to 160 ms in *source 1* and from 167 to 177 ms in *source 2*, corresponding to the typical time course of the LEP N1 component, at the maximum peaks of these two sources the topographic maps were already dominated by the N2 LEP component, maximal over the vertex. Mean values of source dipole moments, time epochs of statistically significant waveform modulations, and *F* and *P* values are shown in Table 2.

There were no effects of session or photograph type on *sources 4* or *5*. Their approximate Talairach coordinates are given in Table 3. There were no statistically significant interactions between sessions and photographs in any of the sources (*P* > 0.05).

#### Correlation data.

After each EEG session, ratings of how enjoyable, filling, and tasty the food in the photographs looked were taken on VAS, along with a rating of how much participants could eat of the different foods if they were hungry. These measures were highly intercorrelated within sessions, so they were collapsed to give a single hedonic rating scale for each session.

To analyze the contribution of hedonism to the activation of *source 3* (right PHG), which showed a statistically significant effect of session, a Pearson's correlation coefficient was computed between hedonic ratings and the source dipole moment during the time interval 248–257 ms (averaged over blocks and photographs). The correlation was significant in the fed session [*r*(11) = 0.625, *P* < 0.05] and stronger during the hungry session [*r*(11) = 0.810, *P* < 0.01]. The correlation coefficients themselves were not significantly different (*P* > 0.05). *Source 3* activation during this epoch was not significantly correlated with pain ratings.

As far as pain rating is concerned, only *sources 1* (operculo-insular cortex) and *6* (cerebellum) were significantly correlated with pain ratings during the time epochs of significant modulation by photograph type. Activation in the operculo-insular cortex was positively correlated with pain ratings in the context of food photographs [*r*(10) = 0.594, *P* < 0.05] and marginally more so in the context of the object photographs [*r*(10) = 0.603, *P* < 0.05]. Activation in cerebellum was markedly more positively correlated with pain ratings in the context of object photographs [*r*(12) = 0.581, *P* < 0.05] than in the context of food photographs [*r*(12) = 0.343, *P* > 0.05]. Again, the correlation coefficients themselves were not significantly different (*P* > 0.05).

## DISCUSSION

Participants reported less pain in the initial block of the experiment and exhibited reduced source activity in right PHG when they were hungry compared with fed; similarly, viewing food photographs resulted in comparatively small but statistically significant reductions in pain-related source activations in left opercular insula, ACC, right PHG, and cerebellum. These results are in agreement with those predicted by the theory of emotion and motivation (Bradley et al. 2001; Lang et al. 1992, 1997) and conform to predictions made by the motivation-decision model (Fields 2007).

Hunger slightly attenuated pain in the first block of the experiment and suppressed activation in the right parahippocampal region. This finding corroborates previous studies that have reported decreased nociceptive processing when fasting (Davidson et al. 1992; de los Santos-Arteaga et al. 2003; McGivern et al. 1979; McGivern and Berntson 1980). A contribution by parahippocampal cortex (or very closely related areas) to evoked pain responses has been postulated previously in EEG source analysis studies (Stancak et al. 2013; Stancak and Fallon 2013; Valeriani et al. 1996, 2000, 2002), and experiments with fMRI have cited PHG as being involved in processes such as reactivating memories of pain (Kattoor et al. 2013), pain anticipation (Fairhurst et al. 2007), and pain sensitivity (Piche et al. 2010). Interestingly, in the present study the effects of session on PHG activation were not correlated with pain ratings but rather with ratings of food photograph hedonicity. Results from appetite research have found PHG to be activated by hunger (Del Parigi et al. 2002; Tataranni et al. 1999) and by appetitive stimuli when participants were fasted (Cheng et al. 2007; LaBar et al. 2001; St-Onge et al. 2005).

Passively viewing food photographs also attenuated pain responses to a small degree in operculo-insular cortex, mid-ACC, PHG, and cerebellum. Insula is reliably activated in response to visually presented appetitive stimuli (Kroemer et al. 2013; Porubská et al. 2006; Simmons et al. 2005; van der Laan et al. 2011) and also forms part of the “pain matrix”; operculo-insular cortex responses to laser-evoked pain are almost universally reported (Garcia-Larrea et al. 2003). This region has been cited as a likely generator of N1 LEPs (Iannetti et al. 2005; Vogel et al. 2003); it is sometimes indistinguishable from secondary somatosensory cortex, since the structures lie so close together and are densely reciprocally connected, and therefore both areas have been implicated as possible generators of N1 in some studies (Frot et al. 1999; Valeriani et al. 2000). The area of ACC identified in the present study is involved in orienting attention to salient stimuli (Kim et al. 2013; Peyron et al. 1999; Tölle et al. 1999), and N1 has recently been demonstrated to be augmented by salient stimuli (Ronga et al. 2013). The timing of the operculo-insular and ACC modulations (150–160 ms and 167–177 ms after the laser stimuli, respectively) by food photographs in the present study corresponds closely to the timing of the classically evoked N1 LEP (García-Larrea et al. 1997; Legrain et al. 2002; Tarkka and Treede 1993; Valeriani et al. 1996), while the peak activations at 188 and 181 ms (respectively) are approaching the usual timing of the N2 LEP component (Friederich et al. 2001; Legrain et al. 2002; Stancak and Fallon 2013). This finding corroborates previous research that has suggested that operculo-insular cortex contributes to both N1 and N2 (Garcia-Larrea et al. 2003; Tarkka and Treede 1993; Valeriani et al. 1996).

N1 has been shown to code pain intensity (Iannetti et al. 2005; Stancak et al. 2012) and, when general vigilance effects are held constant, a change in attentional focus (Legrain et al. 2002). Since participants saw the food photographs before they felt the pain, any reduced operculo-insular and ACC source activation may result from a reduced capacity of the pain to shift attention away from the food.

Right PHG activation was reduced from 300 to 330 ms when participants viewed food photographs (independently of session effects). This corresponds to the usual time frame of the P2 LEP (Friederich et al. 2001; Valeriani et al. 2007; Yamasaki et al. 1999), which appears to reflect the affective processing of pain. Its amplitude can be tempered by viewing pleasing aesthetics (de Tommaso et al. 2008) and pleasant pictures (de Tommaso et al. 2009), and the magnitude of P2 (in the absence of pain) has also been demonstrated to be augmented by emotional words (Herbert et al. 2006; Schacht and Sommer 2009). PHG has also previously been shown to respond to manipulations of emotion in the context of pain (Roy et al. 2009; Stancak et al. 2013), and more generally, as part of the entorhinal cortex, it is often described as being part of an emotion network (see Lindquist et al. 2012 for a comprehensive review). That the PHG activation corresponding to the time frame of P2 was weaker when participants were viewing food photographs suggests that appetitive stimuli might somewhat diminish the affective dimension of pain.

The final area that appeared to be modulated by photograph type was the cerebellum, between 395 and 405 ms. Cerebellum is cited often as a region activated by pain (Moulton et al. 2010; Peyron et al. 2000) and, in conjunction with the limbic structures ACC and PHG, is part of an emotion-related network activated in response to generally aversive stimuli (Moulton et al. 2011). Cerebellum appears to reflect both ascending and descending pain signaling (Borsook et al. 2008; Hagains et al. 2011; Saab and Willis 2003; Yelle et al. 2009); that it was temporally the last structure to participate in the source model of the LEP suggests that it was engaged here in pain inhibition. This explanation is supported by our finding of correlations between pain intensity and the strength of source activity in cerebellum.

MFG formed a statistically important part of the source model, but its activation was not modulated by either appetite status or photograph type. MFG is commonly cited in pain studies (Baron et al. 1999; Brooks et al. 2002; Iadarola et al. 1998; Ochsner et al. 2006; Song et al. 2006; Tracey et al. 2000). It is significantly activated in response to pain regardless of whether the pain is strong or weak (Kong et al. 2010) and is involved in decision making (Krain et al. 2006; Rogers et al. 1999; Schmitz and Johnson 2006) and response selection (Hazeltine et al. 2003; Schumacher et al. 2003). It is therefore not surprising that the experimental manipulations failed to affect activation in MFG, since every trial in every condition required a decision and a response.

The temporal and spatial distribution of experimental effects across varying time intervals is likely due to sequential and hierarchical processing of sensory information, seen also in other sensory modalities (Hari et al. 2010). The N1 component of LEPs, originating primarily in the contralateral operculo-insular cortex and ACC (Fig. 3*B*, *top*), was consistent with an early arrival of nociceptive information via the spinothalamic tract neurons to posterior insula, secondary somatosensory cortex, and ACC, which are the targets of the spinothalamic tract neurons (Dum et al. 2009). The subsequent latency components, in particular the long-latency component peaking at 404 ms (Fig. 3*B*, *bottom*), point to involvement of additional brain regions such as medial temporal cortex, rostral ACC, and cerebellum, consistent with employment of higher-order, top-down control processes at a later stage of nociceptive processing. Therefore, source dipole modeling was required to separate the spatial and temporal components of LEPs.

There was no interaction between sessions and photograph types, which suggests that there may be separate processes contributing to the comparatively small effects on pain. The first (related to session) appears to reflect generally heightened attention to any distracting stimuli when hungry, resulting in a decreased perception of pain and an increased appreciation of the hedonicity of appetitive stimuli. The second (related to photograph type) would be stimulus driven and perhaps manifested as a more direct competition for attention between food stimuli and pain, resulting in slight interference with nociceptive processing.

Our results provide a suggestion as to a possible mechanism underlying previous findings that fasting can reduce pain in chronic pain patients (Michalsen et al. 2002, 2005; Michalsen and Li 2013; Wilhelmi de Toledo et al. 2013), although we acknowledge that the short-lasting experimental pain we employed lacks almost all of the sensory and psychological features commonly seen in chronic pain. Thus the clinical impact of the study is limited and needs to be established in future studies involving chronic pain patients.

### 

#### Limitations.

An additional control condition of pain without the context of any kind of visual stimuli was not included in the study. The extra control would have eliminated any possibility of an effect of unequal attention between the food and object photograph conditions, potentially strengthening our results. However, a control condition not displaying complex pictures may affect nociceptive processing differently from conditions involving pictures of food or objects, because of reduced demand on attention and decreased distraction.

Second, pain ratings were only significantly different for the fed and fasted conditions during the first block; after this they dropped considerably (Fig. 2). The decline in subjective pain ratings is most likely due to habituation. This is an often-cited hazard in LEP research where many trials are required (de Tommaso et al. 2005; Mobascher et al. 2010); one study showed that the habituation effect could be mitigated by continually increasing the laser intensity (Weiss et al. 1997), but this approach was beyond the scope of this study, where it was more important to match the laser intensity across the fed and fasted sessions.

#### Conclusions.

The results of the present study demonstrate that hunger and visual food stimuli may partially suppress the cortical processing of noxious stimuli and induce a short-lived and small reduction in pain intensity. The clinical significance of these findings for pain relief in chronic pain patients is limited at this stage, and will need to be established in future studies.

## GRANTS

This study was supported by the Biotechnology and Biological Sciences Research Council (BBSRC) (grant no. BB/I015809/1).

## DISCLOSURES

No conflicts of interest, financial or otherwise, are declared by the author(s).

## AUTHOR CONTRIBUTIONS

Author contributions: H.W., T.G., A.T., J.A.H., J.C.G.H., and A.S. conception and design of research; H.W., X.L., and N.B.F. performed experiments; H.W. and A.S. analyzed data; H.W. and A.S. interpreted results of experiments; H.W. prepared figures; H.W. drafted manuscript; H.W., X.L., N.B.F., T.G., A.T., J.A.H., J.C.G.H., and A.S. edited and revised manuscript; H.W., X.L., N.B.F., T.G., A.T., J.A.H., J.C.G.H., and A.S. approved final version of manuscript.
